# Induction of chondro-, osteo- and adipogenesis in embryonic stem cells by bone morphogenetic protein-2: Effect of cofactors on differentiating lineages

**DOI:** 10.1186/1471-213X-5-1

**Published:** 2005-01-26

**Authors:** Nicole I zur Nieden, Grazyna Kempka, Derrick E Rancourt, Hans-Jürgen Ahr

**Affiliations:** 1 Molecular & Genetic Toxicology, Bayer HealthCare AG, Wuppertal, Germany; 2 Department of Biochemistry & Molecular Biology, University of Calgary, Calgary, Canada; 3 Faculty of Medicine, Dept. of Biochemistry & Molecular Biology, University of Calgary, HMRB 331, 3330 Hospital Drive NW, Calgary, Alberta, T2N 4N1, Canada

## Abstract

**Background:**

Recently, tissue engineering has merged with stem cell technology with interest to develop new sources of transplantable material for injury or disease treatment. Eminently interesting, are bone and joint injuries/disorders because of the low self-regenerating capacity of the matrix secreting cells, particularly chondrocytes. ES cells have the unlimited capacity to self-renew and maintain their pluripotency in culture. Upon induction of various signals they will then differentiate into distinctive cell types such as neurons, cardiomyocytes and osteoblasts.

**Results:**

We present here that BMP-2 can drive ES cells to the cartilage, osteoblast or adipogenic fate depending on supplementary co-factors. TGFβ_1_, insulin and ascorbic acid were identified as signals that together with BMP-2 induce a chondrocytic phenotype that is characterized by increased expression of cartilage marker genes in a timely co-ordinated fashion. Expression of collagen type IIB and aggrecan, indicative of a fully mature state, continuously ascend until reaching a peak at day 32 of culture to approximately 80-fold over control values. Sox9 and scleraxis, cartilage specific transcription factors, are highly expressed at very early stages and show decreased expression over the time course of EB differentiation. Some smaller proteoglycans, such as decorin and biglycan, are expressed at earlier stages. Overall, proteoglycan biosynthesis is up-regulated 7-fold in response to the supplements added. BMP-2 induced chondrocytes undergo hypertrophy and begin to alter their expression profile towards osteoblasts. Supplying mineralization factors such as β-glycerophosphate and vitamin D_3 _with the culture medium can facilitate this process. Moreover, gene expression studies show that adipocytes can also differentiate from BMP-2 treated ES cells.

**Conclusions:**

Ultimately, we have found that ES cells can be successfully triggered to differentiate into chondrocyte-like cells, which can further alter their fate to become hypertrophic, and adipocytes. Compared with previous reports using a brief BMP-2 supplementation early in differentiation, prolonged exposure increased chondrogenic output, while supplementation with insulin and ascorbic acid prevented dedifferentiation. These results provide a foundation for the use of ES cells as a potential therapy in joint injury and disease.

## Background

Articular cartilage is composed of extracellular matrix (ECM), the matrix-secreting chondrocyte and water, which all account for the tissue's characteristic rigidity as well as its flexibility. These features are necessary in order to warrant life-long survival of the cartilage tissue, especially in the joint where it has to endure pressure forces caused by movement. Chondrocytes arise from a mesenchymal progenitor during development, the same progenitor that gives rise to other mesenchymal cell types including osteoblasts, adipocytes and myocytes. Bone formation can either be endochondral, when chondrocytes mature and calcify to provide a matrix for the invading osteoprogenitors, or intramembraneous involving ossification directly from a mesenchymal ancestor. All these diverse cell types may arise from the same precursor, but are distinguished by specific morphological features and with that, a certain set of characteristic proteins including transcription factors that control their differentiation.

Two chondrocyte-specific transcription factors have been identified, Sox9, a member of the SOX-family of transcription factors, and Scleraxis, a member of the basic helix-loop-helix transcription factors [1,2]. However, most of the exclusive markers for cartilage tissue reside in the ECM. The predominant form of collagen in mature cartilage is collagen type IIB, whereas the alternatively spliced collagen IIA is found primarily during development [3]. Aggrecan is the major proteoglycan species in cartilage [4]. The transition of chondrocytes into hypertrophy is distinguished by a change in expression of Cbfa1, the osteoblast-specific transcription factor, which is also switched on during intramembraneous ossification. Distinctive to cartilage, the major collagen molecule in osseus matrix is collagen type I. In contrast, the transcription factors controlling adipogenesis are C/EBPα and PPARγ, which transactivate subsets of genes as a function of either trans-acting factor alone or requiring the co-operative effort of both [5]. C/EBPα is known to bind to and transactivate particularly the promotors of the SCD1, aP2 and the Glut4 genes [6,7], all highly characteristic of the adipocyte phenotype.

For decades, the treatment of degenerative cartilage and bone diseases has been a challenge for orthopaedic surgeons due to the apparent inability of cartilage and bone to repair itself. Arthritis, a degenerative joint condition, is one of the most prevalent chronic health conditions in North America. Arthritis can devastate people, but to date there is no effective therapy available and patients can only be helped by surgical joint replacement. An inherent major concern is the limited availability of autografts, which significantly reduces the choice of treatable defects. However, new approaches to cell grafting are being developed in this field: increased yields of cells are achieved by the usage of bioreactors and growth factor administration, such as TGFβ_1 _and BMPs [8,9]. Additionally, stem cells are being discovered as a new source of transplantable material.

Embryonic stem cells represent a valuable source for cell transplantation since their characteristic features include an unlimited self-renewing capacity and a multilineage differentiation potential [10,11]. In fact, ES-derived glial precursors and cardiomyocytes have been successfully transplanted, integrated and shown to be functionally active in the transplantation site [12,13]. The yield of differentiation of ES cells into an intended lineage can be greatly enhanced by the addition of growth factors or induction substances. Whereas protocols for the differentiation of cardiomyocytes, neuronal cell types, insulin-producing cells or adipocytes from ES cells have been available for many years [14-17], only recently their differentiation into elements of the skeleton has been reported [18-20]. Our group has previously shown that vitamin D_3 _forces ES cells to undergo osteogenesis [18]. Kramer et al. have reported in 2000 that BMP-2 pushes ES cells to the chondrogenic fate when added during days 3–5 of EB differentiation [21]. Those ES-derived chondrocytes possess a certain plasticity to undergo hypertrophy and calcify [22].

We show here, that prolonged treatment of differentiating ES cell cultures with BMP-2 in synergy with TGFβ_1_, insulin and ascorbic acid leads to improved chondrogenesis *in vitro*. Compared to the brief supplementation described by Kramer et al. [21], the expression of chondrocyte-specific marker genes was highly up-regulated while proteoglycan content revealed an increased chondrocytic yield from 7.26% to 57.03%. As described by other groups [22], ES-derived chondrocytes become hypertrophic and calcify. However, spontaneous calcification did not reach mineralization levels that are found in vitamin D_3 _induced ES-derived osteoblasts [18]. Yet, supplementation of chondrocyte-cultures with β-glycerophosphate, ascorbic acid and vitamin D_3 _starting at day 20 rescued the osteoblast phenotype. In many differentiations, we also observed an accumulation of lipid droplets and an up-regulation of adipocyte-specific genes. This direction towards adipocyte differentiation varied with the use of specific co-factors, suggesting that in the future, such spurious differentiation may be controlled, once the pathways involved in adipogenesis are better understood.

## Results

### Characterization of chondrocyte-like cells derived from ES cells

**Figure 1:**
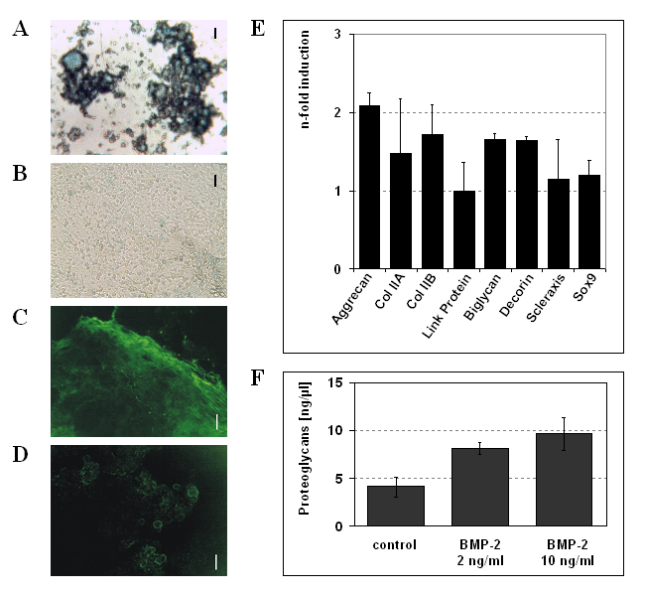
Morphology and characteristics of ES-derived chondrocytes after 32 days of culture. (A, B) Determination of proteoglycans in EBs with alcian blue in chondrocyte cultures induced with TGFβ_1 _[2 ng/ml] and BMP-2 [10 ng/ml] from d3–5 of culture and with BMP-2 [10 ng/ml], ascorbic acid [50 μg/ml] and insulin [1 μg/ml] from day 5 onwards (A) compared to control cultures (B). Bar = 8.3 μm. (C, D) Analysis of cartilage-specific matrix proteins in 32 day old EBs induced with the same supplements as in (A, B) by means of immunohistochemistry. Staining with anti-collagen type II (C) and anti-cartilage proteoglycan (D), respectively, both visualized by a secondary AlexaFluor 488 conjugated antibody. Bar = 106 μm. (E) Concentration-dependent effect of BMP-2 on chondrocyte-specific gene expression in 32-day old EBs. Values of cultures supplemented with 10 ng/ml BMP-2 are shown compared to 2 ng/ml as used by Kramer et al. [2000], which was set as 1. Values represent means of three independent experiments ± standard deviation, obtained by quantitative RT-PCR analysis. **P < 0.01; ***P < 0.001. (F) Proteoglycan content of EB extracts on day 32. BMP-2 directs increased proteoglycan synthesis in both 2 ng/ml and 10 ng/ml. *P < 0.1; **P < 0.01. Mean ± standard deviation, n = 3.

Embryonic stem cell cultures supplemented with BMP-2, TGFβ_1_, insulin and ascorbic acid show typical morphological changes compared to the untreated cultures. Starting with the fourth week of culture, aggregates consisting of small round cells formed in the supplemented cultures, which stained positive with alcian blue (fig. 1A). Little alcian blue staining was seen in control cultures (fig. 1B). Polygonal cells, which could also be found in treated cultures, did not stain with alcian blue. Significant immunostaining for the collagen type II (COL II) protein was observed at day 32 in treated cultures corresponding to the active secretion and formation of an extracellular matrix found with chondrocytes. The COL II antibody identified the fibrillary organization of the collagen molecules in the extracellular matrix (fig. 1C). Chondrogenic differentiation was confirmed by positive immunostaining for adult proteoglycans (fig. 1D), which is detectable in the aggregates identified by alcian blue staining. The distribution was associated with the extracellular matrix similar to that found with the COL II antibody. Staining appeared to be diffuse as extracellular matrix and not individual cells are stained.

### Dependence of gene expression patterns and proteoglycan synthesis on BMP-2

Quantitative RT-PCR was used to examine the variability of RNA expression of various cartilage-specific genes in response to BMP-2. Previously, chondrogenesis was induced in ES cells using 2 ng/ml BMP-2 on days three to five of EB formation, when early mesodermal markers such as *Brachyury *and *BMP-4 *are expressed [21,23,24]. To improve chondrogenesis, we investigated whether a higher concentration of BMP-2 (10 ng/ml) could increase chondrocyte-specific gene expression. Total RNA was extracted on day 32 and quantitative real-time PCR was carried out. Expression of genes of interest in cultures supplemented with 10 ng/ml BMP-2 was normalized to GAPDH expression and compared to cultures supplemented with 2 ng/ml (fig. 1E), which were set as 1. Expression of the small leucine-rich proteoglycans biglycan and decorin was increased 1.6 fold upon supplementation with 10 ng/ml BMP-2 (P < 0.01). Neither link protein expression nor Sox9 or scleraxis expression were affected by the higher dosage. However, expression of aggrecan and the collagen type II isoforms A and B, which are specific for mature chondrocytes, were significantly increased compared to the low BMP-2 concentration described by Kramer et al. (P < 0.001). The degree of chondrogenic differentiation under influence of BMP-2 was further quantified by metachromatic detection of proteoglycans (fig. 1F). Secreted proteoglycans were extracted on day 32 of culture with guanidine/HCl and combined with dimethylmethylenblue. Based on our measurement of aggrecan, the synthesis of proteoglycan proteins is also increased under the influence of BMP-2. BMP-2 at 2 ng/ml initiated an induction in proteoglycan synthesis of about 2 fold (P < 0.01), whereas 10 ng/ml BMP-2 increased the proteoglycan content of EBs 2.3 fold (P < 0.1) compared to non-treated controls. This data shows, that BMP-2 causes the induced synthesis of negatively charged extracellular matrix, characterized by proteoglycans.

### Additive effect of TGFβ_1_, insulin and ascorbic acid on BMP-2 induced chondrogenesis

**Figure 2:**
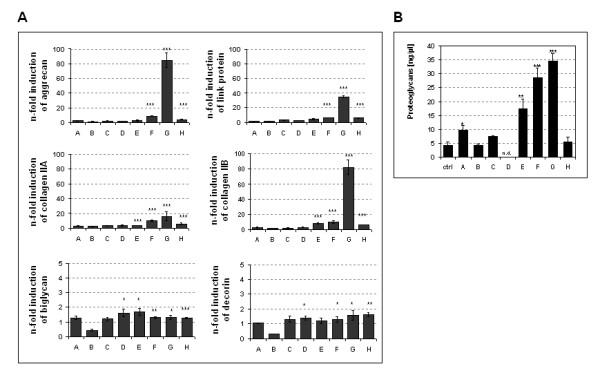
Changes on cartilage markers in response to various combinations of BMP-2, TGFβ_1_, insulin and ascorbic acid as outlined in table 1. (A) Diagram of expression niveaus of cartilage-specific genes of 32 day old EBs obtained by quantitative RT-PCR and standardized to GAPDH. Untreated control values were set as 1. Mean ± standard deviation, n = 3. *P < 0.1; **P < 0.01; ***P = 0.001. (B) Proteoglycan content of extracts of 32 day old EBs treated with different combinations of BMP-2, TGFβ_1_, insulin and ascorbic acid (see table 1). Ctrl = control. Means ± standard deviation, n = 3. *P < 0.1; **P < 0.01; ***P = 0.001. n.d. = not determined.

Since BMP-2 is believed to play a role in late chondrogenesis [25], we studied the effect of prolonged BMP-2 supplementation beyond day 5 of culture. Additionally, the anabolic effect of insulin and ascorbic acid and the influence of the growth factor TGFβ_1 _on BMP-2 induced differentiation was determined quantitatively by PCR analysis at various stages throughout EB differentiation. Table 1 shows the particular combinations of medium supplements used at different culture stages of the 'hanging drop' protocol used for differentiation, in which day 1–3 represent the hanging drop stage. During days 3–5 the resulting embryoid bodies are cultured in suspension and then plated onto tissue culture treated plastic ware on day 5. Applied concentrations were 10 ng/ml BMP-2, 2 ng/ml TGFβ_1_, 1 μg/ml insulin and 50 μg/ml ascorbic acid. Dexamethasone, which is also known to be a chondro-inducing agent [26], did not evoke a mentionable chondrogenic response in the D3 ES cell line (data not shown). Figure 2 shows changes in cartilage-specific gene expression under the influence of various differentiation co-factors throughout the 32 days of culture. TGFβ_1 _(combination B) evoked a 2-fold increase in collagen II expression for both splice forms. The synergistic effect of both growth factors, TGFβ_1 _and BMP-2 (combination C) began to show an increased expressions of aggrecan, link protein and COL IIA compared to cultures that were treated with one supplement alone. Additional supplementation with insulin and ascorbic acid between culture days 3 and 5 (combination D) barely increased aggrecan, link protein and COL II expression, but when given from day 5 onwards enhanced both the BMP-2 and TGFβ_1 _effects (supplement combinations E and H). Addition of insulin and ascorbic acid to BMP-2 induced cultures increased collagen type IIA and aggrecan expression minimally to 1.2-fold, link protein and collagen type IIB were induced 2.7- to 2.8-fold compared to BMP-2 alone. The TGFβ_1 _response was increased 3- to 4-fold. When cultures were induced with BMP-2 and TGFβ_1 _in suspension (d3–5) and supplemented with insulin and ascorbic acid starting on day 5 onwards (combination F), aggrecan, link protein and COL II were up-regulated 10-fold compared to controls.

**Table 1:** Combinations of medium supplements used at different culture stages

**Supplement combination**	**Day 3–5 (hanging drops)**	**Day 5 onwards (attached culture)**
**A**	BMP-2	
**B**	TGFβ_1_	
**C**	BMP-2 TGFβ_1_	
**D**	BMP-2 TGFβ_1_ insulin ascorbic acid	
**E**	BMP-2	insulin ascorbic acid
**F**	BMP-2 TGFβ_1_	insulin ascorbic acid
**G**	BMP-2 TGFβ_1_	insulin ascorbic acid BMP-2
**H**	TGFβ_1_	insulin ascorbic acid

Applied concentrations were 10 ng/ml BMP-2, 2 ng/v ml TGFβ_1_, 1 μg/ml insulin and 50 μg/ml ascorbic acid. The end of the culture period varied with every experiment carried out.

Surprisingly, when BMP-2 supplementation was maintained throughout the culture period (combination G), a dramatic increase of marker gene expression was seen. Aggrecan and COL IIB were up-regulated over 80-fold above controls and link protein and COL IIA expression was increased to 35- and 16-fold, respectively. Here, scleraxis and Sox9 expression behaved conversely. Supplement combination G showed significant decreases of scleraxis and Sox9 expression to 50 and 87% of the control, respectively (P = 0.001). Deferral of the expression of these transcription factors in favour of chondrogenic differentiation characterizes BMP-2 induced differentiation, whereupon chondrogenic processes are furthermore enhanced by TGFβ_1_, insulin and ascorbic acid.

The determination of proteoglycan content was used to confirm the results obtained by quantitative RT-PCR for all supplement combinations (fig. 2B). TGFβ_1 _alone (combination B) did not alter proteoglycan levels compared to controls, and in combination with BMP-2 decreased the proteoglycan content of EBs compared to BMP-2 alone (combination C). Insulin and ascorbic acid did not enhance the proteoglycan synthesis in combination with TGFβ_1_, but caused a 4.2-fold increase in combination with BMP-2 (combination E, P = 0.01). In agreement with aggrecan gene expression, combinations F and G generated a massive 7-fold induction of proteoglycans in EBs (P = 0.001).

**Figure 3:**
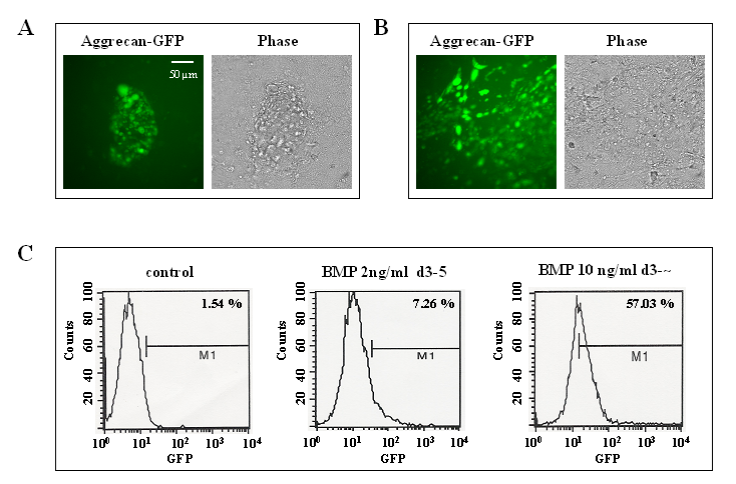
Quantification of chondrogenic yield by flow cytometry. (A, B) Genetically modified ES cells expressing GFP from the chondrocyte-specific aggrecan promotor. Green fluorescing chondrocytes appear as clusters of cells within the remaining cell population (A), but are also scattered in the entire population starting at day 28 of differentiation (B). (C) FACS analysis of ES-derived chondrocytes sorted by their GFP expression. Differentiation with BMP-2 only [2 ng/ml, d3–5] produced 7.26% GFP expressing cells compared to spontaneously differentiated controls, which contained very low levels of fluorescing cells (1.54%). Prolonged BMP-2 administration together with TGFβ_1_, ascorbic acid and insulin (supplement combination G, see table 1) raised chondrocyte outcome to 57.03%.

Genetically manipulated ES cells that express GFP under the control of chondrocyte-specific aggrecan promotor were then used to quantify chondrocyte yield using fluorescence-activated cell sorting. ES cells were differentiated along the chondrocytic lineage using BMP-2 at 2 ng/ml or supplement combination G [d3–5: TGFβ_1 _10 ng/ml, BMP-2 10 ng/ml; d3–32: BMP-2 10 ng/ml, ascorbic acid 50 μg/ml and insulin 1 μg/ml]. Green fluorescing chondrocytes in both cultures appeared either organized in clusters or scattered as seen in figure 3A. The Kramer protocol gave a 7.26 percent yield of chondrocytes, which was increased to 57.03% using our modified protocol (fig. 3B).

### Kinetic analysis of cartilage-specific gene expression during EB differentiation

**Figure 4:**
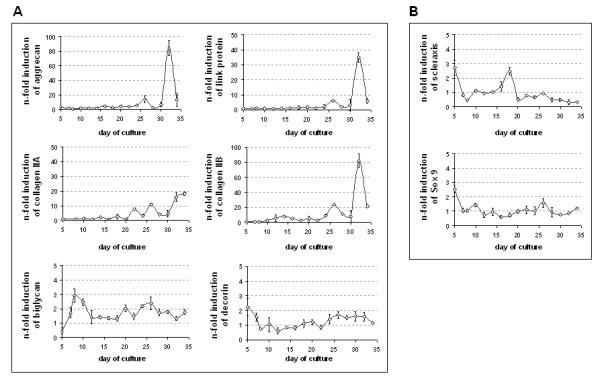
Expression of cartilage-specific genes in the course of EB differentiation induced by BMP-2, TGFβ_1_, insulin and ascorbic acid (supplement combination G, table 1). (A) Aggrecan, collagen type II A and B and link protein, biglycan and decorin. (B) Scleraxis and Sox9. Results show induction factors of expression obtained by quantitative RT-PCR and normalized to GAPDH expression in comparison to corresponding spontaneously differentiated controls, which were set as 1 (mean ± standard deviation, n = 3 independent experiments, 20 EBs each).

The degree of chondrogenic differentiation using BMP-2, TGFβ_1_, insulin and ascorbic acid supplementation was then examined over the 35-day culture period using quantitative RT-PCR of various cartilage matrix genes (fig. 4). Since the extent of changes in cartilage-specific genes seemed to be dependent on acute (d 3–5) or chronic (d 3–32) application of BMP-2 (supplement combination F versus supplement combination G), the stronger induction by chronic supplementation of BMP-2 was monitored throughout the culture duration. During the first three weeks of the BMP-2 induced differentiation, only minor changes in aggrecan, link protein or collagen type II expression could be detected. Starting with day 26 however, aggrecan expression was up-regulated significantly to 14-fold over control niveaus (P < 0.1). Reaching day 32, aggrecan, link protein and collagen type II A and B approached peak levels. Consistent with the aggrecan expression profile, link protein expression was found to be significantly up-regulated on days 24–27 (factor 6.3; P = 0.001). Increased values were detectable as early as day 16. On day 26, the quantification of the collagen type IIA and B showed 11- and 24-fold increases respectively. On day 14, splice form B had already reached an 8-fold increase over control values. In contrast, during the early phases of differentiation, between day 5 and 20, collagen type IIA expression was continuously increased 2-fold. Transcripts for biglycan and decorin were detectable throughout all stages of chondrogenic differentiation. With the beginning of the maturation phase in the fourth week of culture (day 21–28), both genes were up-regulated 1.5–2 fold. Transcription factors scleraxis and Sox9 (fig. 4B) showed a similar expression profile constantly over the entire culture period. This level did not change significantly during chondrogenic differentiation, but rather decreased compared to controls. Both were already transcribed in day 5 EBs, hallmarking their participation in early differentiation events, where lineage specificity is determined. Additionally, scleraxis transcripts were engaged in later phases of development between days 16–19. Sox9 expression was also increased between days 9–11 and between days 25–27. In conclusion, cultures supplemented with BMP-2, TGFβ_1_, insulin and ascorbic acid express mRNAs of a chondrogenic phenotype, whose expression was time-dependent.

### ES-derived chondrocytes undergo hypertrophy and mineralize

**Figure 5:**
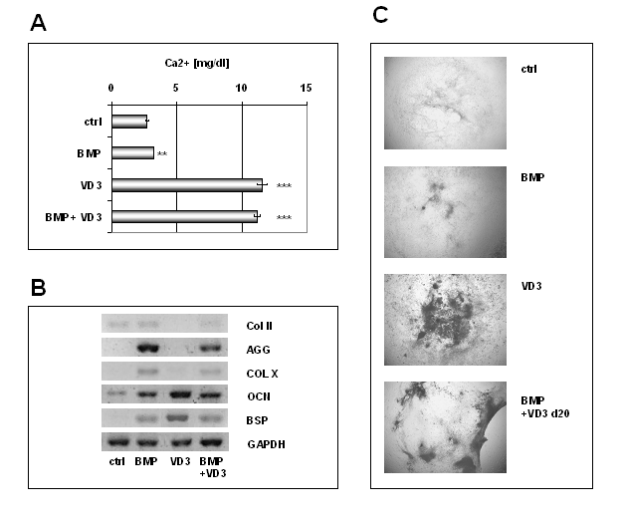
Degree of osteogenesis in cultures treated with chondroinducing medium (BMP, supplement combination G), osteoinducing medium (VD_3 _as described in ref. 18) and control ES medium (ctrl). Osteoblast phenotype and mineralization could be increased in chondrocyte cultures by adding VD_3 _to chondroinducing supplements at day 20 (BMP+VD_3_). (A) Extent of mineralization as measured by Ca^2+ ^content. **P < 0.01; ***P = 0.001. (B) RT-PCR for chondrocyte-specific and osteoblast-specific marker genes as well as Collagen X as a marker for the hypertrophic state of chondrocytes. (C) Morphology as seen in phase contrast. Black appearing cells were identified as being mineralized osteocalcin expressing osteoblasts previously [18]. No such cells can be seen in control (ctrl) and chondrocyte (BMP) but in osteoblast (VD_3_) and rescued osteoblast cultures (BMP+VD_3_).

During embryo development endochondral ossification occurs in two steps: chondrocytes arise after mesenchymal condensation and become hypertrophic, characterized by expression of collagen type X, and calcification. To test whether this was true for the ES-derived chondrocytes generated with our protocol, we assayed for the Ca^2+ ^content of the cultures, a measure for mineralization (fig. 5A). Calcium was increased 1.2-fold in cells that were supplemented with BMP-2, TGFβ_1_, insulin and ascorbic acid compared to controls (P < 0.01). Previously, we have described the induction of mineralization in osteoblasts derived from ES cells with vitamin D_3_, which reach maturity at day 32 of culture [18,27]. Here, we observed that the level of mineralization was considerably higher in direct osteoblast differentiation [11.57 mg/dl] compared to indirect differentiation using our improved chondrocyte protocol [3.22 mg/dl]. We observed, however, that the level of calcium found in VD_3 _induced ES-derived osteoblasts could be rescued in the chondrocytes by adding VD_3 _on day 20 of differentiation, a time when chondrocytes could be morphologically identified in the cultures (11.18 mg/dl, P < 0.001). As shown by quantitative RT-PCR, VD_3 _treated ES cell derived chondrocytes could alter their expression profile to that of d32 ES cell derived osteoblasts (fig. 5B). However, expression of osteocalcin and bone sialoprotein was less than in VD_3 _rescued chondrocyte cultures than in VD_3 _osteoblast cultures. Interestingly, chondrocyte-specific genes could not be detected in VD_3 _osteoblasts and waned in VD_3 _rescued chondrocytes. As we have shown previously, ES-derived mineralized osteocalcin expressing osteoblasts can be identified as black appearing cells in phase contrast microscopy [18]. Figure 5C shows these black cells in the VD_3 _treated cultures. No such cells are visible in control cultures or in ES-derived chondrocyte differentiations. However, by adding VD_3 _back in at day 20 to the chondrocytes, mineralization can be detected, although the localization pattern is slightly different. These observations support the hypothesis that VD_3 _induces intramembraneous bone formation directly from mesenchymal progenitors while BMP-2 controls endochondral bone formation.

**Figure 6:**
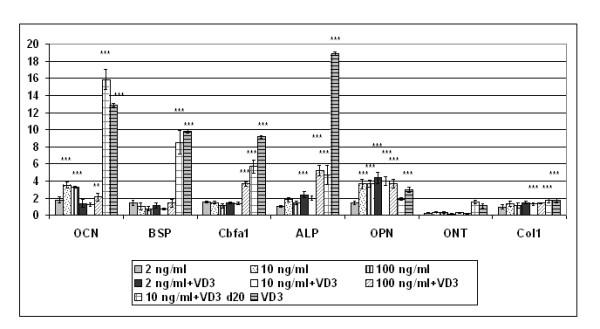
Dependence of genes expressed in cartilage tissue on BMP-2 with and without vitamin D_3 _(VD_3_). All cultures contained β-glycerophosphate [10 mM] and ascorbic acid [50 μg/ml]. Mean ± SD of independent triplicates was quantified by qPCR and is normalized to GAPDH expression. Controls were set as 1. **P < 0,01; ***P = 0,001. OCN = osteocalcin, BSP = bone sialoprotein, Cbfa1 = Core binding factor alpha, ALP = alkaline phosphatase, OPN = osteopontin, ONT = osteonectin, Col1 = Collagen type I. Concentrations of BMP-2 up to 100 ng/ml do not lead to osteoblast-specific expression levels that are reached with VD_3 _only. 100 ng/ml BMP-2 plus VD_3 _given together early during differentiation can significantly up-regulate osteoblast genes. However, when VD_3 _is administered later (d20) in combination with BMP-2, osteoblast gene expression is almost restored.

It has been shown by others that the BMP-2 induced alteration in cell fate is both concentration- and time-dependent [28]. Lower concentrations of BMP-2 support chondrogenesis whereas higher concentrations promote osteogenesis. In ES cells, BMP-2 at concentrations of 2 ng/ml, 10 ng/ml and 100 ng/ml did not increase the expression of bone markers with the exception of osteocalcin and osteopontin, which were significantly increased as shown by quantitative RT-PCR (fig. 6). In combination with VD_3 _(given on days 5–30) however, alkaline phosphatase and Cbfa1 were also significantly up-regulated above controls (5.4- and 4-2-fold, respectively, P < 0.001). As we noted earlier, BMP-2 induced osteogenesis with or without VD_3 _supplementation did not meet the levels that were attained by VD_3 _alone, but the late addition of VD_3 _on day 20 rescued bone-specific gene expression arguing for an involvement for BMP-2 in endochondral bone formation.

### Induction of adipocyte differentiation

**Figure 7:**
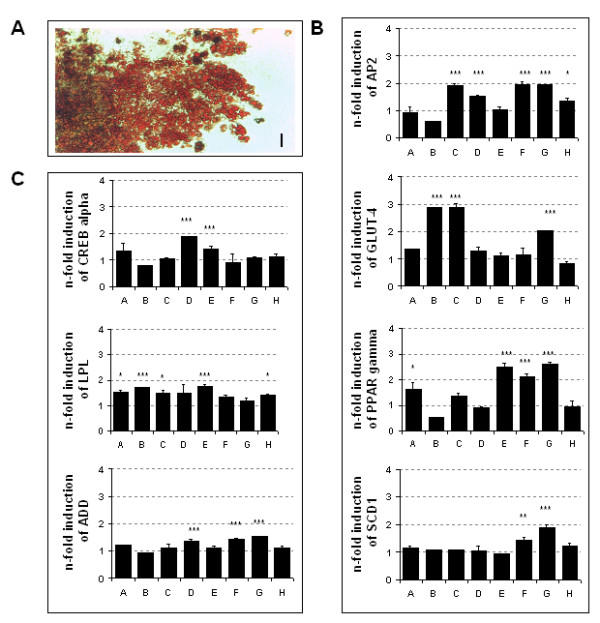
Characterization of ES-derived adipocytes. (A) Oil-Red-O staining of lipid droplets in ES-derived adipocytes. Bar = 42 μm. (B, C) Influence of BMP-2 alone and in combination with TGFβ_1_, insulin and ascorbic acid (see table 1) on expression of adipocyte-specific genes. Quantitative RT-PCR was performed on 30 day old EBs. Expression of genes of interest was normalized to GAPDH and compared to untreated controls. Values show means ± standard deviations on n = 3 independent experiments. *P < 0.1; **P < 0.01; ***P = 0.001.

During treatment of EBs with various chondrocyte differentiation inducing factors, we noticed accumulation of lipid droplets, which could not be found in untreated controls. Those droplets could indeed be characterized as lipid-containing by means of Oil-Red-O staining (fig. 7A). Quantitative real-time PCR analysis revealed a slight up-regulation of adipocyte-specific genes (ADD1, aP2, C/EBPα, GLUT-4, LPL, PPARγ and SCD1) on day 30 in most of the supplement combinations used for induction of chondrocyte differentiation (fig. 7B,C). Supplement combination G, which was the most successful for inducing chondrocyte differentiation suppressed adipocyte differentiation. GLUT-4 was most up-regulated in combinations B and C, whereas combinations E and B induced a increase in LPL expression compared to our chondrocyte differentiation protocol.

## Discussion

In this study, we demonstrate an improved method for driving ES cells to a cartilaginous fate when stimulated with BMP-2 and TGFβ_1_. In the early phase of differentiation, BMP-2 operates by directing differentiation towards the cartilage lineage and acting on chondroprogenitors. During later differentiation adding mineralizing agents can trigger hypertrophy and mineralization of the ES-derived chondrocytes. In the embryo, maturation of chondrocytes in the process of endochondral ossification follows a timely regulated developmental program, whereby cellular stages can be delimitated molecularly. During ES cell differentiation into chondrocytes, developmental processes follow the same pattern, as judged by the gene expression patterns observed. Chondrocytes, osteoblasts and adipocytes are thought to arise from the same mesenchymal progenitor. Based on our previous observations around VD_3_-induced osteogenesis, we have already hypothesized that during the first 5 days of differentiation mesodermal progenitors develop, which then are susceptible to the VD_3 _treatment [18]. Treatment of the cultures with TGFβ_1 _at days 3–5 may augment the number of mesenchymal progenitors, as TGFβ_1 _is thought to inhibit the proliferation of most cells, but to stimulate some mesenchymal cells such as osteoblasts and chondrocytes [8]. It was not surprising to see adipocytes develop in many of our cultures, as they represent another member of the TGFβ_1 _promoted mesodermal lineage.

The gain of adipose characteristics in culture is hallmarked by: a) the appearance of cytoplasmic lipid droplets, b) the acquisition of insulin sensitivity with regard to glucose uptake (GLUT4) and c) the expression and secretion of numerous bioactive molecules [5]. All of these characteristics of *in vivo *adipogenesis were met in the EBs treated with BMP-2, TGFβ_1_, insulin and ascorbic acid. Indeed, a future challenge for improving adipogenic cultures will be the discovery of regulatory pathways in adipogenesis. Once identified, such pathways may be antagonized in order to enhance ES differentiation into chondrocytes.

During the revision of this paper, a study was published describing that the overexpression of the sox triad, namely Sox9, Sox5 and Sox6, markedly increased chondrocyte marker gene expression (collagen type 2, aggrecan) in ES cells within 3 days [29]. Here, TGFβ_1_, BMP-2, IGF-1 and FGF-2 had no affect on the immediate regulation of these genes. In our differentiation system, Sox9 is elevated very early during differentiation at day 5 (data not shown) underlining its role as an early controller of chondrogenesis. We have shown here that BMP-2 regulates later processes in cartilage development. Marker gene expression levels reached upon overexpression of the Sox trio do not meet the levels we have observed in this study, suggesting that Sox9 may be necessary but not sufficient do direct all progenitors to the chondrocytic lineage.

Earlier this year, another study portrayed chondrogenesis in ES cells using encapsulation in alginate [26]. The usage of a 3D culture system led to an increase in Col II and aggrecan expression of about 1.5- to 2-fold above the regular 2D system of plating EBs. Comparing those values to the 80- to 90-fold up-regulation described here, it is clear that three-dimensional signals also need to be incorporated into chondrocyte differentiations to increase differentiation efficiency.

Articular cartilage has been refractory to repair following degeneration. Despite its limited capacity to self-repair, cartilage is replete with cells capable of undergoing mitotic division [30]. Current hypotheses suggest that cells may be constrained by their ECM, thus preventing expansion and differentiation or that there is a limit in the bioactive molecules, which support chondrogenesis [31,32]. Engineering bone or cartilage usually requires the handling of autologous cells. Cells are released from the ECM using collagenase and hyaluronidase. However, the small number of available progenitors, within the tissue can be problematic [33]. Moreover, the ability of these harvested cells to proliferate is limited in the elderly, where most degenerative joint disorders occur. The outcome of conventional surgical treatment including joint resurfacing or biological autografts has been unsatisfactory following long-term evaluation [34]. This failure is caused by insufficient repair resulting in the formation of mechanically inadequate resident fibrocartilage. These disappointing results and the limited therapeutic opportunities have led investigators to focus on more appropriate bioregenerative tissue engineering approaches, which could be specifically tailored for a patient's needs.

Pluripotent ES cells are now being contemplated as a new cell source for tissue engineering since they are the most multifaceted cells amongst all stem cells. While their pluripotency offers a huge potential for cell therapies directed against a wide spectrum of degenerative diseases that are ineffectively treated by traditional approaches, it also poses challenge for controlling developmental fate in the recipient. Understanding the developmental pathways regulating ES cell differentiation, will enable the creation of a cell source that can be manipulated to correct for a particular defect.

This study was designed to improve upon the number of differentiated cells needed for the *in vitro *development of functional cartilage as this represents a first critical step in applying ES cells clinically. Additionally, the presented *in vitro *model of chondrogenesis may be useful for *in vitro *embryotoxicity tests [27] as also serve as a new tool in identifying molecular pathways that underlie chondrogenesis. Compared to other *in vitro *models of chondrogenesis, this model incorporates all steps of development beginning with a pluripotent, uncommitted cell and thus might further our understanding of normally unamendable stages of development.

## Conclusions

This study was particularly designed to improve chondrocyte yields from ES cells by investigating the effect of higher BMP-2 concentrations and the complementary effects of TGFβ_1_, insulin and ascorbic acid. By examining gene expression responses and cell sorting for GFP-expressing chondrocytes, we demonstrate that using a higher concentration of BMP-2 in combination with appropriate co-factors, we can significantly enhance ES cell derived chondrogenesis compared to known protocols. In addition, we document that ES-derived chondrocytes behave naturally as they undergo hypertrophy.

We show that EBs supplemented with BMP-2 also result in a small amount of adipogenesis *in vitro*. This observation is consistent with knowledge that adipocytes, chondrocytes and osteoblasts arise from the same mesenchymal ancestor. Accordingly, in the future, it will be necessary to understand how to discriminate these populations for cytotherapeutic applications. At this time, the presented *in vitro *model allows the study of mechanisms involved in BMP-2 induced chondrogenesis, osteogenesis and adipogenesis.

## Methods

### Cell culture and differentiation of embryonic stem cells

Cells of the mouse ES cell line D3 (American Type Culture Collection, Rockville, Maryland, USA) were kept in permanent culture as described [35,18] with the additive Leukemia Inhibitory Factor (1000 U/ml, Gibco Life Technologies, Karlsruhe, Germany). Differentiation was initiated in hanging drops. Cells condensed to form EBs, which were transferred on day 3 into suspension culture. At day 5, EBs were plated into 24-well tissue culture primaria plates (Falcon, Heidelberg, Germany). The effects of BMP-2 [2 or 10 ng/ml], TGFβ_1 _[2 ng/ml], insulin [1 μg/ml] and ascorbic acid [50 μg/ml] in various combinations on the differentiation of chondrocytes were examined. Medium was changed every second day.

### Alcian blue staining

Proteoglycans secreted by ES-cell derived chondrocytes were stained with Alcian blue. Cultures were fixed in 2.5% glutaraldehyde, 25 mM sodium acetate, 0.4 M MgCl_2 _containing 0.05% Alcian blue for 48 h. Wash steps in 3% acetic acid, 3% acetic acid/25% ethanol and 3% acetic acid/50% ethanol reduced unspecific binding of the dye.

### Metachromatic test with 1.9-dimethylmethylenblue

Proteoglycan content of differentiated cultures was determined with the DMMB-assay [36]. Proteoglycans were extracted in 4 M guanidin-HCl/0.05 M sodium acetate (pH 5.8) containing 100 mM 6-amino-caproic acid, 10 mM EDTA, 5 mM benzamidine/HCl, 10 mM N-ethylmaleimide, 0.4 mM pepstatin, 1 mM PMSF and 1 μg/ml soy bean trypsin inhibitor for 48 h at 4°C. Non-completely digested cells were separated from the lysate by centrifugation. Lysate was mixed with DMMB reagent (0.16% w/v DMMB in 0.2% formic acid containing 2 mg/ml sodium formate, pH 3.5) and changes in absorption were detected at 535 nm in a spectrophotometer. Concentration of proteoglycans in samples was read against a standard curve of chondroitin sulfate C.

### Immunofluorescence

Embryoid bodies were differentiated to the chondrocyte lineage with BMP-2, TGFβ_1_, insulin and ascorbic acid and fixed on day 32 with ice-cold methanol:aceton (7:3) at -20°C for 10 min. For staining with anti-collagen type II (Chemicon, MAB 8887) cultures were digested with pepsin for 15 min at 37°C. Staining with anti-adult proteoglycan (Chemicon MAB 2015) was performed after a 1 h digest with chondroitinase ABC at room temperature. Cells were overlaid with the appropriate dilution of the first antibodies in PBS, 10% FCS at 4°C over night. The corresponding secondary antibody, an AlexaFluor 488 goat anti-mouse IgG (H+L), F(ab')_2 _fragment (A-11006, Molecular Probes, Leiden, The Netherlands) was incubated with the cells for 2 h at room temperature. Cultures were observed in a Leica Fluovert FU fluorescent microscope (Leitz, Wetzlar) with an excitation wavelength of 495 and an emission wavelength of 519 nm.

### RNA isolation, RT-PCR and real-time quantitative RT-PCR

Total RNA was isolated from 20 EBs per probe using the RNeasy Midi Kit (Qiagen, Hilden, Germany) according to the manufacturer's instructions with on-column DNase I digestion. The amount of RNA was determined using the RiboGreen™ RNA quantitation reagent and kit (Molecular Probes). 275 ng total EB RNA was used as a template for cDNA synthesis with Superscript II (Invitrogen, Paisley, Scotland) as described [27,35] in a total reaction volume of 25 μl. 5 μl aliquots of the first strand reaction were used for amplification performed with specific primers (see table 2). Primer sequences for osteoblast-specific genes and PCR conditions have been described previously [18]. PCR products were visualized on 3% agarose gels containing 0.1 μg/ml ethidium bromide. Quantitative real-time PCR analysis was performed in an ABI Prism^® ^7700 Sequence Detector. The accumulation of reaction products during PCR was monitored by measuring the increase in fluorescence caused by the binding of SYBR^® ^Green (Applied Biosystems, Perkin Elmer, Weiterstadt, Germany) to double-stranded DNA. Expression analysis of collagen type II A and B was done in the same PCR run with two differently labelled probes specific for either the A isoform (5'-CGAGATCCCCTTCGGAGAGTGCTGT-3'/VIC) or the B isoform (5'-CCAGGATGCCCGAAAATTAGGGCCAA-3'/FAM) [27]. Reaction mixtures were set up as suggested by the manufacturer containing either SYBR Green or probes for collagen type II. Following a 10 min Taq Polymerase activating step at 95°C, the reactions were cycled by denaturing at 94°C for 30 s and annealing and elongation for 30 s at the corresponding temperatures (table 2). Target gene C_T_-values were standardized against GAPDH expression and induction of expression in treated EBs was normalized to control EBs. Primer sequences for murine GAPDH were 5'-GCACAGTCAAGGCCGAGAAT-3' and 5'-GCCTTCTCCATGGTGGTGAA-3' (T_a _= 60°C).

**Table 2:** Sequences and annealing temperatures for the primer sets used in RT-PCR

**Gene**	**Primer sequence**	**T_m _in °C**
**Chondrocyte-specific**		
Aggrecan	5'-GATCTGGCATGAGAGAGGCG-3' 5'-GCCACGGTGCCCTTTTTAC-3'	61
Collagen type II	5'-GCTGCTGACGCTGCTCATC-3' 5'-GGTTCTCCTTTCTGCCCCTT-3'	60
Collagen type X	5'-CAAGCCAGGCTATGGAAGTC-3' 5'-AGCTGGGCCAATATCTCCTT-3'	60
Link protein	5'-TTCTGGGCTATGACCGCTG-3' 5'-AGCGCCTTCTTGGTCGAGA-3'	60
Biglycan	5'-CATGACAACCGTATCCGCAA-3' 5'-ATTCCCGCCCATCTCAATG-3'	60
Decorin	5'-ATGACCCTGACAATCCCCTG-3' 5'-CCCAGATCAGAACACTGCACC-3'	60
Scleraxis	5'-GGACCGCAAGCTCTCCAAG-3' 5'-ACCCACCAGCAGCACATTG-3'	62
Sox9	5'-GCAGACCAGTACCCGCATCT-3' 5'-CTCGCTCTCGTTCAGCAGC-3'	62
**Adipocyte-specific**		
ADD1	5'-CAGTGACTCTGAGCCCGACA-3' 5'-ATGCCTCGGCTATGTGAAGG-3'	61
PPARγ	5'-ATCATCTACACGATGCTGGCC-3' 5'-CTCCCTGGTCATGAATCCTTG-3'	59
SCD1	5'-ACACCATGGCGTTCCAAAAT-3' 5'-CGGCGTGTGTTTCTGAGAACT-3'	61
C/EBPα	5'-CGCAAGAGCCGAGATAAAGC-3' 5'-GCGGTCATTGTCACTGGTCA-3'	60
GLUT-4	5'-ATGGCTGTCGCTGGTTTCTC-3' 5'-ACCCATAGCATCCGCAACAT-3'	59
AP2	5'-TGATGCCTTTGTGGGAACCT-3' 5'-GCAAAGCCCACTCCCACTT-3'	58
acrp30	5'-AAGAAGGACAAGGCCGTTCTC-3' 5'-GAGGAGCACAGAGCCAGAGG-3'	60
LPL	5'-CCAATGGAGGCACTTTCCAG-3' 5'-CCACGTCTCCGAGTCCTCTC-3'	60

Forward primer sequence is depicted from the 5' to the 3' end followed by the reverse primer. The appropriate annealing temperature for each set is given as T_m _in °C.

### Quantification of chondrocyte yield by FACS

The GFP expressing plasmid pEGFP-1 (Clontech) under the control of the Aggrecan promotor (kind gift of John R. Matyas) was stably transfected into D3 ES cells using Invitrogen's Effectene system followed by neomycin selection. Genomic integration of the reporter was analyzed by RT-PCR with specific GFP primers (data not shown). ES cells were differentiated along the chondrocyte lineage, trypsinized into a single cell suspension and subjected to fluorescence-activated cell sorting on day 32 using a FACS Calibur instrument and the CellQuest software from Becton Dickinson (Germany). Ten thousand events were registered per sample and analysis of whole cells was performed using appropriate scatter gates to avoid cellular debris and aggregates.

### Oil-Red-O staining

Cells were washed with PBS and without any fixation directly overlaid with Oil-red-O working solution (0.18% Oil-Red-O dye/60% propanol) After a staining period of 15 minutes, ES cell cultures were rinsed in distilled water until desired colour was achieved.

### Statistical analysis

Data analysis was performed with ONE-WAY ANOVA on n = 3 experiments using SigmaStat version 2.03 (SPSS Inc., San Rafael, CA, USA).

## List of abbreviations

ADD1 Adipocyte determination- and differentiation-dependent factor 1

aP2 Fatty acid-binding protein

BMP Bone Morphogenetic Protein

Cbfa1 Core binding factor alpha 1

C/EBP CCAAT/enhancer-binding protein

Col Collagen

DMMB Dimethylmethylenblue

ECM extracellular matrix

ES embryonic stem

EB embryoid body

FCS Fetal calf serum

FGF Fibroblast growth factor

GAPDH Glyceraldehyde-3-phosphate dehydrogenase

GLUT4 Glucose transporter 4

IGF Insulin-like growth factor

PPAR Peroxisome proliferator-activated receptor

SCD Steroyl CoA desaturase

Sox Sry-related high mobility group box

TGFβ Transforming Growth Factor beta

VD_3 _Vitamin D_3_

## Authors' contributions

NZN carried out cell culture, biochemical and molecular studies and drafted the manuscript. GK, DER and HJA participated in its design and coordination. All authors read and approved the final manuscript.
